# Overcoming the underdiagnosis of obstructive sleep apnea to empower genetic association analyses

**DOI:** 10.1093/sleep/zsac312

**Published:** 2022-12-15

**Authors:** Tamar Sofer

**Affiliations:** Division of Sleep and Circadian Disorders, Brigham and Women’s Hospital, Boston, MA, USA; Department of Medicine, Harvard Medical School, Brigham and Women’s Hospital, Boston, MA, USA; Department of Biostatistics, Harvard T.H. Chan School of Public Health, Boston, MA, USA

Genome-wide association studies (GWAS) of complex traits, that is, phenotypes that are influenced by many genetic variants, have discovered thousands of genetic loci [1] underlying blood pressure, diabetes, lipids, psychiatric, and other traits, including sleep-related phenotypes [2]. However, studies of obstructive sleep apnea (OSA) have been less fruitful in that fewer discoveries have been made. Two major factors have limited OSA GWAS. First, few epidemiologic studies have measured OSA, and those that did so often studied a subset of participants rather than the full sample of large cohort studies, resulting in low sample sizes for GWAS (in comparison with other phenotypes) [3–6]. Second, while the availability of large biobanks that collected genotyping data in conjunction with electronic health records, including the UK Biobank (UKB), FinnGen, and Biobank Japan, accelerated GWAS of many phenotypes, including OSA [7], challenges lingered because OSA is underdiagnosed [8, 9]. As a result, many individuals with OSA are misclassified as “controls”. Thus, while current estimates of OSA prevalence in the United States are around 17% in women and 34% in men [10], and similarly, high prevalence is reported elsewhere, the prevalence of OSA status in the UKB is only about 1% [11] and about 8% in FinnGen [7] (gender combined).

Misclassification of OSA reduces the power to discover genetic associations and biases effect-size estimates, in a manner depending on the OSA prevalence and on the misclassification rate. Figure 1 provides a schematic tabulation of the true OSA status compared to the OSA status observed in a population. Out of *n*_1s_ = *n*_10_ + *n*_11_ individuals with OSA in the population, *n*_11_ individuals are indeed observed to have OSA, and *n*_10_ individuals appear to have no OSA, despite having OSA. Define the misclassification rate as π= *n*_10_/*n*_1s_, the proportion of individuals with OSA who are erroneously classified. Using the same notation, the prevalence of OSA in the healthcare system or study is *n*_1s_/(*n*_1s_ + *n*_0s_) = *n*_1s_/*n*. I performed a simulation study to demonstrate how misclassification of OSA may bias genetic effect estimates and reduce power (see https://github.com/tamartsi/OSA_misclassification for code). Using a simple logistic regression model, I assumed that OSA probability depends on a population-based constant, the intercept $\beta_{0}$ (which may be thought of as the average of many factors, including genetic ones), and on a single-modeled genetic variant g via the standard logistic model equation:

**Figure 1.**
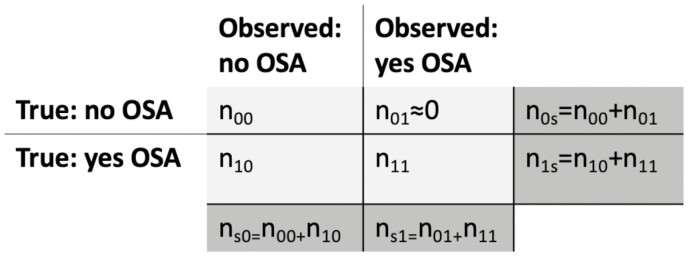
True OSA status versus observed OSA status. Tabulation of the observed OSA status against the true, underlying OSA in a given study. The number of individuals in the study is decomposed into individuals in each of the table cells. The marginal, dark gray, cells sum the individuals in the rows and in the columns. In health records, typically we expect that the number of individuals who in truth do not have OSA yet are observed as having OSA is near zero.


$$\begin{array}{l} logit\left(Pr\left(OSA=1\right)\right)=\beta_{0}+g\times \beta_{g}. \end{array}$$

The simulations had $\beta_{g}$, the log odds ratio (OR), set to 0.1, corresponding to an OR of 1.10, while $\beta_{0}$ took the values −1.5, −1, and −0.5, corresponding to true underlying OSA prevalence of about 19%, 28%, and 39%. The genetic variant g was sampled from a binomial distribution with probability 0.3 and a count of 0, 1, or 2, representing a genetic allele with frequency 0.3 across two chromosomes. Using the equation above, in each iteration of the simulation OSA probability was computed, and next true OSA status was sampled from the resulting probability. The next step induced misclassification, where individuals with true OSA = 1 had observed OSA with probability 1 − π. Misclassification rate took the values 0.4, 0.6, and 0.8. For context, if the true OSA population prevalence in the UKB and FinnGen is 25%, their misclassification rates are 96% and 68%, respectively. The simulations iterated 1000 times for each combination of true OSA prevalence and misclassification rate, with a total sample size of *n* = 20 000 in each simulation iteration.

Table 1 provides the simulation results. Indeed, the power to detect the association of the genetic variant with OSA is reduced as the misclassification rate is increased: for a modest OSA prevalence of about 19%, a misclassification rate of π=0.4 results in 0.76 power while with π=0.8, the power is reduced to 0.29. When the true OSA prevalence is higher, the power is higher (when using both the true and the misclassified OSA). Yet, even with a true OSA prevalence of 39%, with π=0.8, the power is still very low at 0.37. Further, the estimated variant effect size is reduced toward the null as the misclassification rate increases, with a higher reduction when the true OSA prevalence is higher.

**Table 1.** Simulation results demonstrating bias and power loss caused by misclassification of OSA cases

π Misclassification rate	Mean estimated $\beta_{g}$ (true OSA)	Mean estimated $\beta_{g}$ (observed OSA)	Bias of $\beta_{g}$ estimates (observed OSA)	Power (true OSA)	Power (observed OSA)
**True OSA prevalence 19%**					
** 0.4**	0.100	0.090	0.010	0.96	0.76
** 0.6**	0.100	0.086	0.014	0.96	0.57
** 0.8**	0.099	0.081	0.019	0.95	0.29
**True OSA prevalence 28%**					
** 0.4**	0.100	0.086	0.014	0.99	0.84
** 0.6**	0.099	0.080	0.020	0.98	0.65
** 0.8**	0.101	0.077	0.023	0.98	0.38
**True OSA prevalence 39%**					
** 0.4**	0.099	0.079	0.021	0.99	0.86
** 0.6**	0.100	0.071	0.029	0.99	0.64
** 0.8**	0.099	0.065	0.035	1.00	0.37

For each combination of parameters determining OSA prevalence and its rate of misclassification, the simulations compare the estimated effect size (log odds ratio) when using the real OSA status and when using the observed OSA status, that suffers from misclassification, as mean estimates across 1000 simulation repetitions. The power is computed as the proportion of simulations in which the *p*-value of the genetic variant effect estimate was <.05.

To address the reduced power caused by OSA misclassification, Campos et al. [12] performed a multi-trait analysis, combining OSA GWAS with a GWAS of snoring, and discovered 49 loci associated with OSA, snoring, or both. Multi-trait analyses have been used to discover genetic associations with other trait groups, including blood pressure, anthropometric, psychiatric traits, and others [13–15]. Such approaches are limited in that identified genetic associations cannot be attributed with confidence to any one trait. Importantly, Campos et al. [12] addressed this limitation via an OSA-specific replication analysis. They replicated 29 of the 49 discovered associations in a BMI-adjusted OSA GWAS in 23andMe, which had an OSA prevalence of ~11%. This suggests that the 29 replicated loci are indeed associated with OSA, and not only with snoring. This replication rate is higher than the replication rate reported when using US-based healthcare systems to estimate the genetic association of variants that were reported in OSA-focused studies with substantially smaller sample sizes [16].

The principle of leveraging genetic associations with OSA-related traits to discover OSA-specific genetic associations is useful. It could be extended to excessive daytime sleepiness (EDS), the most common presenting symptom of OSA [17], to insomnia, as we recently found that a polygenic risk score of insomnia is associated with OSA [18], and to other OSA-associated phenotypes. However, it remains important to validate associations with OSA in independent studies, and preferably in studies that correctly classify OSA cases and controls (as much as possible given the variability in OSA indices such as the apnea–hypopnea index [19]).

As shown in Table 1, the misclassification of OSA results in biased genetic effect estimates. The simulated example is simplistic, as it assumes that OSA misclassification does not depend on the genetic variant. In reality, it is expected that misclassification will be more or less severe depending on the mechanism underlying the genetic variant’s association with OSA, and how it manifests in other phenotypes. OSA is heterogeneous, and some OSA subtypes manifest in higher daytime sleepiness or other symptoms [20, 21], leading to higher likelihood of diagnosis. The study of Campos et al. may have better captured genetic variants corresponding to OSA subtypes that also manifest in snoring.

Knowledge about the specific OSA consequences associated with the variant can be leveraged, with the development of an appropriate statistical method, to compute unbiased effect size estimates for the variant-OSA association. Figure 2 shows a directed acyclic graph where a genetic variant g is known to be associated with EDS, with an estimated odds ratio ${OR}_{g}^{eds}.$ In a given population, it should be possible to estimate the association of OSA with EDS: ${OR}_{osa}^{eds}$. Assuming that g is associated with EDS only via its effect on OSA, that is, OSA completely mediates the association of g with EDS, one should be able to “reverse” the standard mediation analysis to estimate ${OR}_{g}^{osa}$. Whether such an estimate will be more accurate than an estimate of variant–OSA association obtained in a small study with unbiased OSA classification, is a topic that warrants further statistical and empirical research. Nonetheless, obtaining more accurate estimates of OSA effect sizes, that are not biased by OSA misclassification, is important for downstream applications such as Mendelian randomization analysis.

**Figure 2.**
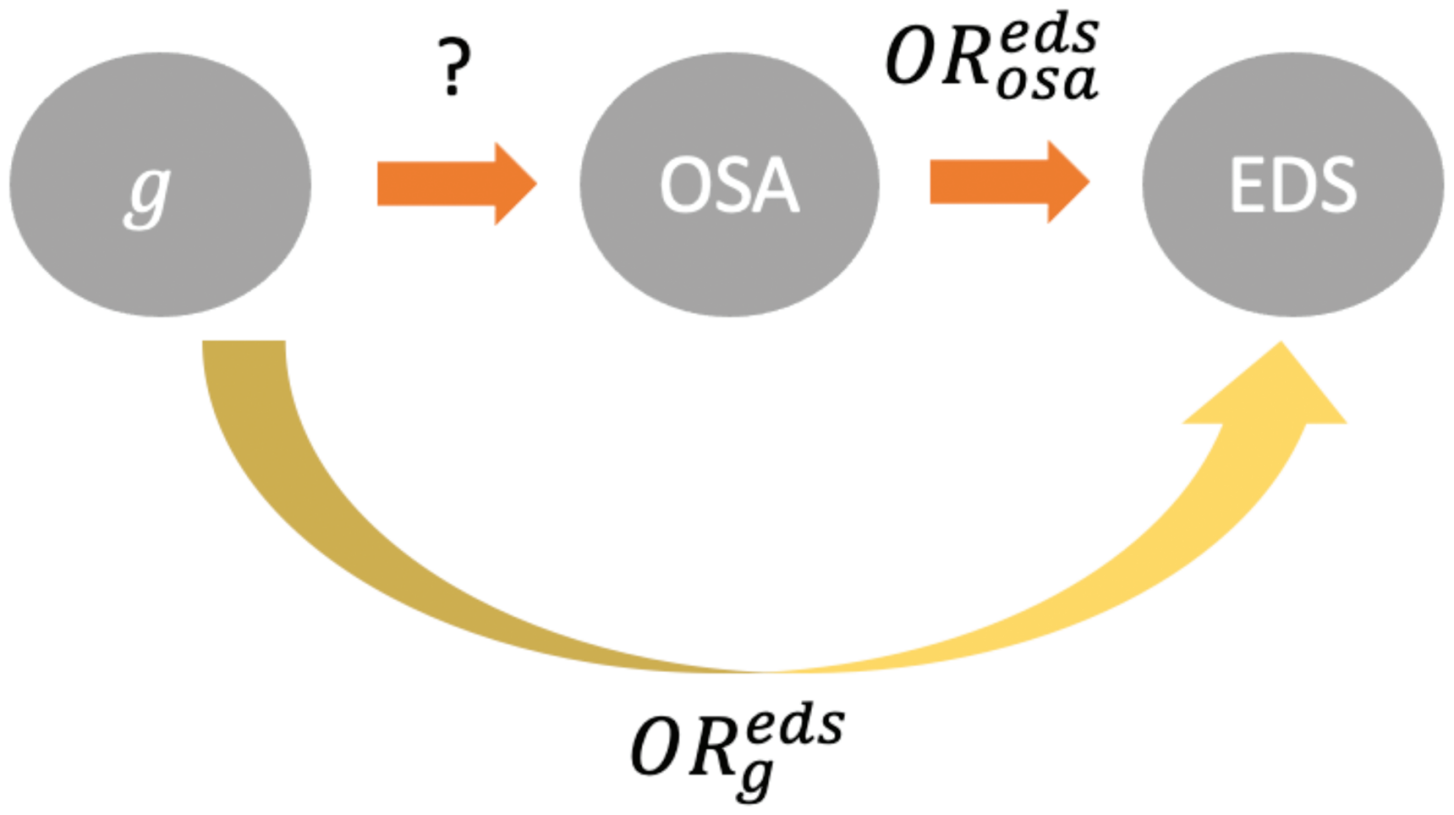
Directed acyclic graph connecting OSA, excessive day time sleepiness, and a genetic variant. The directed acyclic graph presents a potential mediation relationship between a genetic variant, OSA, and excessive daytime sleepiness (EDS). Assuming that the effect of g on EDS is only mediate through OSA, given appropriate methodology one can use the estimated association of g  with EDS ${OR}_{g}^{eds}$and the estimated association of OSA with EDS ${OR}_{osa}^{eds}$ to estimate ${OR}_{g}^{osa}$.

## Data Availability

No data were analyzed in support of this manuscript.
